# A Self-Induced Foreign Body in the Urinary Bladder of an Adolescent Female

**DOI:** 10.7759/cureus.61811

**Published:** 2024-06-06

**Authors:** Hafeez Sohaib Ahmad Warraich, Zirwa Younis, Jawairia Warraich, Aurangzab Shaukat Ali, Khizra Warraich

**Affiliations:** 1 Urology, Allied Hospital Faisalabad, Faisalabad Medical University, Faisalabad, PAK; 2 Obstetrics and Gynaecology, Allied Hospital Faisalabad, Faisalabad Medical University, Faisalabad, PAK; 3 Radiology, Allied Hospital Faisalabad, Faisalabad Medical University, Faisalabad, PAK; 4 Internal Medicine, Madinah Teaching Hospital, Faisalabad, PAK

**Keywords:** pelvic imaging, dysuria, cystoscopy, intravesical foreign body, child and adolescent

## Abstract

In the pediatric population, foreign bodies within the urinary bladder are uncommon, typically resulting from urethral insertion out of curiosity. Other etiologies include sexual assault, iatrogenic factors, or migration from adjacent sites. Symptoms such as urinary retention, dysuria, increased frequency, decreased volume, nocturia, hematuria, painful erections, and pelvic pain are common. Radiographic imaging in the form of pelvic X-rays, ultrasound and CT scans often aids in diagnosis and making an action plan. Management depends on the object type, size, location and available expertise, often starting with a transurethral approach and resorting to open surgery if necessary. This case report describes a 13-year-old female presenting with severe dysuria and visible hematuria. Initially reporting the accidental insertion of a scarf pin into her vagina, she later admitted to intentionally inserting it. A pelvic radiograph revealed a needle-like object in the pelvis but its location and position were more convincing of its presence in the urinary bladder. A diagnostic cystoscopy was performed which confirmed a scarf pin in the urinary bladder, embedded in its wall. The pin was successfully removed transurethrally using endoscopic forceps.

## Introduction

Foreign bodies in the lower genitourinary tract represent an unusual urological emergency [1]. The bladder is the most frequent site where such foreign objects are found [2]. This phenomenon is more prevalent among patients aged 10-20, but cases involving older individuals have been reported more recently [3]. Males are approximately twice as likely to present with this condition compared to females [3]. The reasons for this occurrence can be diverse, encompassing exotic impulses, psychological issues, sexual curiosity, assault, or sexual abuse [1].

In children, foreign bodies within the urinary bladder are quite rare, typically resulting from insertion through the urethra due to curiosity [4]. Other possible causes in both children and adults include sexual assault, iatrogenic factors, or migration from adjacent sites [4]. A wide variety of objects have been reported as foreign bodies in the urethra and bladder, including pencils, telephone cables, thermometers, glass rods, toothbrushes, candles, fruit pits, fish hooks, drinking straws, nails, rifle bullets, chewing gum, snakes, razor blades, wrist watches, and batteries [2].

Symptoms associated with foreign bodies in the lower urinary tract include urinary retention, dysuria, increased urinary frequency, decreased urine volume, nocturia, hematuria, painful erections, and pain in the urethra or pelvis [2]. Due to embarrassment, patients might delay seeking medical attention [1]. Long-term complications from foreign bodies can involve urinary tract infections (UTIs), hematuria, increased urinary frequency, dysuria, urethral false passages, strictures, fistulas, and pain [5].

Diagnosis of a foreign body in the lower urinary tract is based on a comprehensive approach that includes patient history, physical examination, urinalysis, and imaging studies. Urinalysis may reveal red blood cells or pus, suggesting trauma or infection. Radiographic imaging, such as X-rays, often provides visual confirmation of radiopaque foreign objects [1].

Management strategies for foreign bodies in the lower urinary tract vary depending on the type of object, its location, the surgeon's expertise, and the available instruments [2]. Typically, a transurethral approach is the first course of action but if this method is unsuccessful, open surgery may be required [3]. In either case, prompt surgical intervention is critical to prevent complications and ensure optimal patient outcomes.

## Case presentation

**Figure 1:**
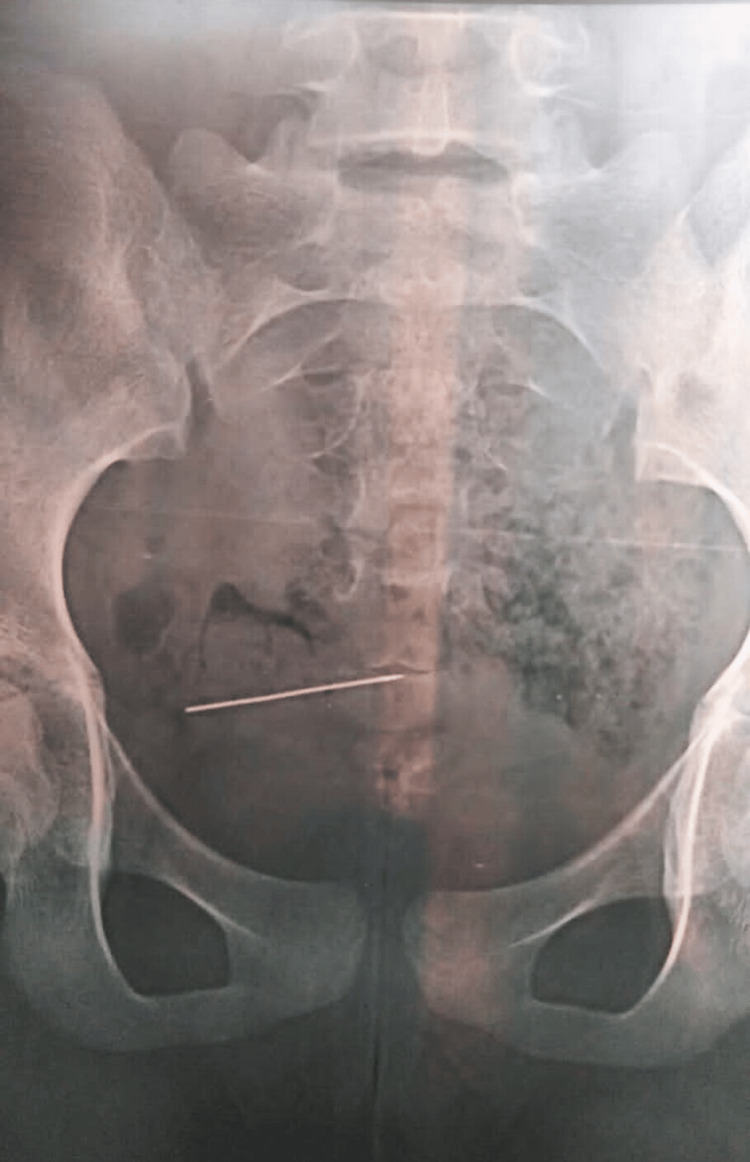
A pelvic radiograph in an anteroposterior view A straight needle is present in the pelvic cavity.

A 13-year-old female with severe dysuria and visible hematuria was referred to the urology department, Allied Hospital Faisalabad by the Department of Obstetrics and Gynecology of the same hospital as an emergency case. The adolescent initially gave the history of accidental entry of a scarf pin into her vagina while scratching the introitus because of itching but later she admitted to a deliberate attempt to insert the pin for curiosity reasons two days ago. A pelvic radiograph was ordered by the on-call gynaecologist (Figure 1), which showed the presence of a straight needle inside the pelvis but the location and position of the needle were more convincing of its presence in the urinary bladder.

**Figure 2:**
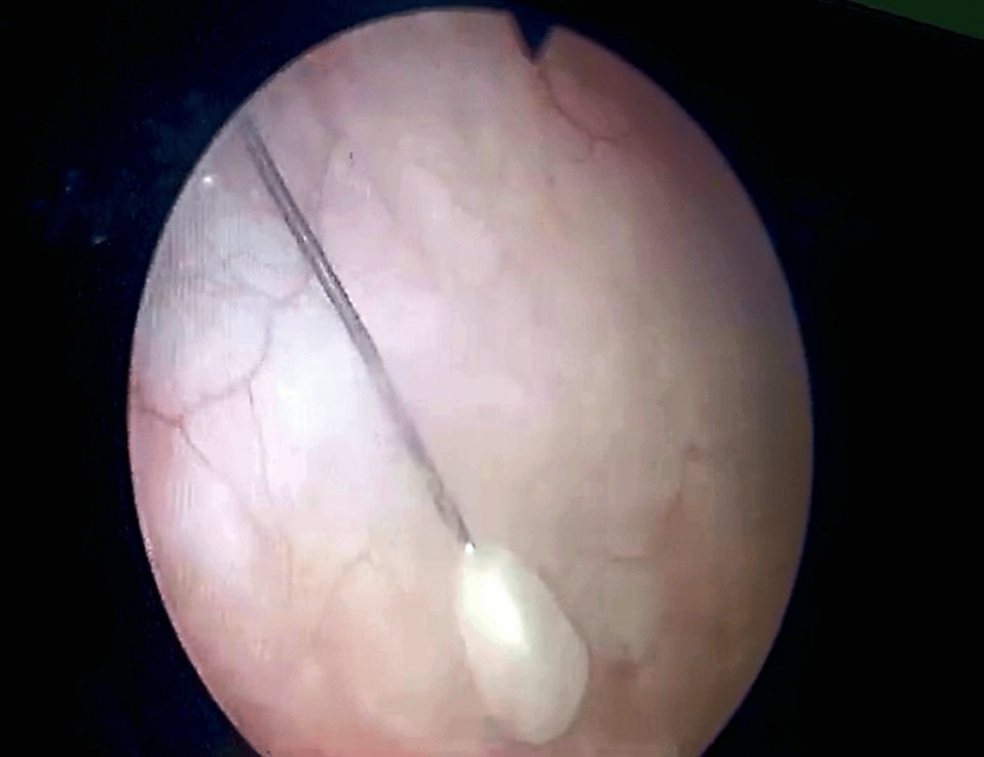
A cystoscopic view of the needle

Digital examination per vagina was not performed because her parents were reluctant to consent to that. A urine complete examination showed a field full of red blood cells and pus cells. The general physical examination and the systemic examination were unremarkable. Because of the position of the needle on the pelvic radiograph and also because of associated dysuria and hematuria, the opinion of the urologist was requested. She was immediately shifted to the urology ward and was admitted there with a plan of diagnostic cystoscopy. A Foley catheter was passed and the irrigation with normal saline was started to prevent intravesical blood clot formation and urinary retention. The next day cystoscopy was performed that confirmed the presence of a scarf pin inside the urinary bladder (Figure 2). One end of the pin had a rounded plastic bead on it while its sharp end was piercing the bladder wall near the base of the bladder and was firmly embedded in it.

**Figure 3:**
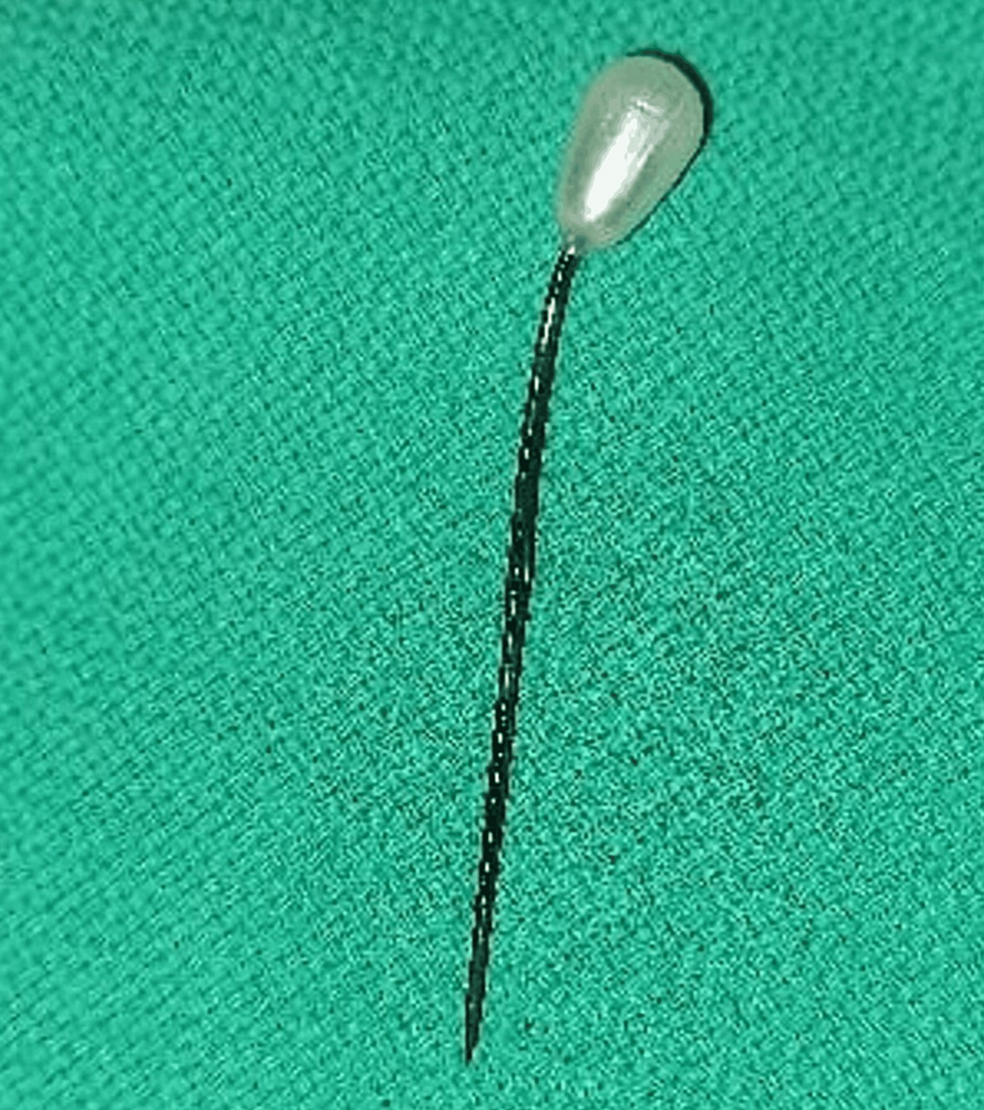
The retrieved needle along with its terminal plastic bead

After confirming these findings, a double J stent removing forceps was passed into the bladder and the pin was pulled out from the bladder wall. Once dislodged, its sharp end was grasped between the two prongs of the forceps and the pin was retrieved out of the bladder through the urethra (Figure 3). A Foley catheter was passed and was removed after a few hours when hematuria was settled.

Postoperative recovery remained uneventful and the patient was discharged on the first postoperative day on oral antibiotics and analgesics, and the parents were counselled regarding the psychological evaluation of the adolescent.

## Discussion

The presence of foreign objects in the urogenital tract is an uncommon pathological occurrence, yet it presents significant urological challenges in terms of diagnosis and management [6]. Foreign bodies in the urinary bladder are particularly rare and can be difficult to diagnose and treat [7]. In children and adolescents, these occurrences might result from sexual abuse, although the inquisitive nature of children can also lead to foreign bodies entering the urinary bladder. This age group may delay seeking medical help due to ignorance or threats from abusers, creating additional responsibilities for clinicians who must be alert to both psychological and medico-legal aspects.

Delayed presentation of foreign bodies in the urinary bladder can complicate the management plan due to secondary complications such as bladder stones, cystitis, chronic obstruction-induced bladder wall hypertrophy, hydronephrosis, renal function impairment, and, in extreme cases, bladder wall perforation.

Diagnosis relies on patient history and clinical examination, with additional imaging studies such as ultrasound, X-rays, CT scans, and MRI to aid in identifying and locating foreign bodies. In some emergencies, a simple radiograph may not provide adequate information for precise localization, and advanced imaging could be contraindicated due to radiation exposure or cost considerations. In such circumstances as in this case, cystoscopy serves as both a diagnostic and therapeutic tool, enabling direct visualization and potential extraction of the foreign body [8].

Endoscopic interventions are a common and effective approach in urological practice to remove intravesical foreign bodies. This procedure, performed using a cystoscope, allows visualization and precise extraction of the foreign body using a variety of instruments, including baskets, forceps, clamshells, and grasping tools. While cystoscopic extraction is generally successful, its efficiency can vary, with reported success rates ranging from 50% to 90% [9]. In cases where a foreign body in the bladder has become calcified over an extended period or is too large, an endoscopic approach might be insufficient, necessitating an open surgical procedure to remove the object [10]. The need for open surgery underscores the importance of early detection and intervention to minimize complications and optimize patient outcomes.

## Conclusions

To conclude, it is still very rare to witness a child or adolescent with an intravesical foreign body. However, when presented, the initial history could be misleading, necessitating careful consideration of the psychological aspects. It is pivotal to retrieve the foreign body urgently to prevent the occurrence of complications. Endoscopic modalities are both diagnostic and therapeutic and are effective in most cases but rarely open surgery may also be required. All such cases should also be considered for psychological evaluation.
